# Everybody's Page

**Published:** 1890-03-22

**Authors:** 


### Everybody's Page.

Nothing is lost in ivory manufactures. Scrapings and sawdust are burnt to charcoal and form the "ivory black" of the artist.

Apropos of St. Patrick's Day, the shamrock used by the Irish is said to have been introduced by St. Patrick as a simile of the Trinity; and the trefoil has been supposed erroneously to have received the name of shamrock in contradistinction to the true rock of the church, St. Peter.

Scents are said to have had an enormous sale in the reign of George the Fourth, so much so that at last the use of them was considered quite vulgar. To many people the smell of any scent is distasteful, and many of the perfumes used now-a-days are so strong as to be sickening.

Those who regard infant insurance, burial clubs, and other similar Prudential institutions as sure signs of thrift and industry, may like to know that some of those among the industrial classes who disapprove of any such clubs give them the euphonious name of "The Wish-Me-Dead Club." Parliament is thinking of protecting further the lives of children who are sent out to nurse. The insured children, we think, want a trifle more done for them, too; if not, why the use of this opprobrious term?

Periodically, a laugh and a most necessary warning are raised about the prevailing use of spectacles among the young, and sad though it is to see a bit of a girl with glasses perched on her diminutive nose, it is also ludicrous. Dr. Collineau and others have recommended regular inspection of eyesight, but so much might be done if parents and teachers would take a little more care that the light of the schoolroom was neither too glaring nor too dull, that the type of books was good, and that the child sat without stooping with his work squarely before him.

There is no country in the world which has a milder or more even climate than the South-west of England, and yet every year people who are in real or imaginary ill-health, come to the conclusion that they can't "winter in England," and go off to the South of France for some months, and return to execrate England and its climate, and to extol the beautiful blue of the Mediterranean. Is this all because of the climate? The thermometer says distinctly "no." To a large number of the frequenters of the South of France, the excitement, the fashion, the gaming tables are the attraction. Yet to others, the genial pleasant life and intercourse with all sorts of people, which, alas, are so lacking in our English health resorts, are no small inducement to them to flit.

That wealth is any enemy to health seems a contradictory statement at first sight, but, briefly considered, it shows itself unfortunately to be anything but that. The making of money keeps a man occupied and healthy, but it is when the fortune is accumulated that the mischief begins. The worn out old rhyme that "Early to bed and early to rise, keeps a man healthy, wealthy, and wise" has a great deal of truth in it, for we naturally conclude that the man who takes the trouble to "rise" early, would scarcely do so unless he meant to fill up the time thus gained by giving exercise to his physical and mental powers, and to this species of individual wealth does no harm. But when, having striven and accumulated, a man sits down to take life easily, which means very often luxury, self indulgence and diminished activity of mind and body, wealth is distinctly an enemy to long life and health, not only to the one generation, but to those who follow after, and who suffer from the sins of their fathers unto the third and fourth generation.

The decrease of pauperism in Scotland, concurrently with an increase of population, is of happy augury for the future.

The Jews in San Francisco show an unsectarian spirit which is worthy of imitation all the world over. They are the most generous supporters of the largest charities in the City, and support even such a distinctively Christian Institution as the Young Men's Christian Association.

What is more delicious to eat than a good ripe apple? and German analysts will tell us that its refreshing taste is only one of its minor qualities. Apples contain a larger amount of phosphorous than any other fruit or vegetable. Here is an opportunity for those who are working at high pressure, and want to renew their brain power.

Electrical execution has been abused and praised in the same way that everything new is criticised; but one thing in its favour is that death by this means is sure. Hanging has been proved to be not always so sure. The minimum time which it takes to effect death is said not to have been accurately determined at the recent experiments; but Dr. Fell opened the windpipe of a calf the instant it had received the electrical current, and kept up forced respiration for half-an-hour all to no effect. So this is fair proof that death *is* instantaneous.

The brick-making trade is seriously threatened in Hamburg! A paper house has been built in that city, impervious to fire in the inside and to wet on the outside. Prejudice is ridiculous, and paper houses may be very comfortable, but we confess they sound a little fragile. An Englishman's house is his castle; at least, there is some worn-out old saying to that effect, whatever it may mean, and a paper castle would scarcely give the feeling of security. "Looking backward," our descendants may probably sneer at our bricks and mortar—who knows?

The *Journal d'Hygiène* once more warns people of delicate digestive power and the public generally of the dangers of ice. Sometimes what with hearing of tubercle in meat or trichinosis in pork, the advantages of this method of taking meals against the disadvantages of the other, the boiling or non-boiling of milk, and so on *ad infinitum*, we feel we must either starve or eat everything without question and risk the consequences. But ice really is a luxury, and none but the best ought to be used. Americans, who use such gigantic quantities of it, are said to be about to introduce severe penalties to prevent the sale of impure ice, for freezing does not destroy every sort of bacteria. People are apt to forget that when water is bad the ice formed from it is also bad.

Automatic machines of all kinds are found in every conceivable place, but they are not played out yet, if report be true. A Dutch chemist, as we reported some months ago, has invented an automatic doctor. This being a somewhat startling announcement, we will explain that he is a dwarf who is pierced with slots all over his body. If you have a pain in your liver, you put a coin into the slot which is in the liver of this silent Æsculapius, and so for all other ailments; wherever your pain you put your money in the corresponding part, and are instantly provided with a small bottle of medicine or a box of pills. Many will exclaim on the danger of such an automaton; is it more dangerous than the silly practice of the crowds of people who are never happy unless they are dosing either themselves or their children with patent medicines of all kinds? On the whole we prefer the new doctor.

